# Supplementary material for: Effects of Chronic Mild Stress on Cardiac Autonomic Activity, Cardiac Structure and Renin–Angiotensin–Aldosterone System in Male Rats
Source: Vet Sci. 2022 Sep 29;9(10):539. doi: 10.3390/vetsci9100539 (PMC9611573; doi:10.3390/vetsci9100539)
Supplement: Supplementary file 1 [file vetsci-09-00539-s001.zip › vetsci-1853240-supplementary.pdf]

Supplementary information

Supplemental Method

Table S1. Overview of the weekly CMS procedure.

Day	Stressors
Sunday	15:00 h: Food deprivation for 18 h
Monday	09:00 h: Food restriction (1/4 pellet, approximately 2.0-2.5 g) for 1 h
	16:00 h: Wetted cage (500 ml water per 500 g of corn cob bedding) for 21 h
Tuesday	10:00 h: Tilting of the cages (45°) for 3 h
Wednesday	15:00 h: Water deprivation for 18 h
Thursday	09:00 h: Empty bottle was given for 1 h
Friday	13:00 h: Group housing (4 rats/cage) for 2 h
	15:00 h: Water deprivation for 18 h
Saturday	09:00 h: Empty bottle was given for 1 h
	18:00 h: The light was turned on overnight, thus the rats were exposed to continuous light for 36 h.

Supplemental Results

Figure S1. Daily food intake during 28-day CMS period.

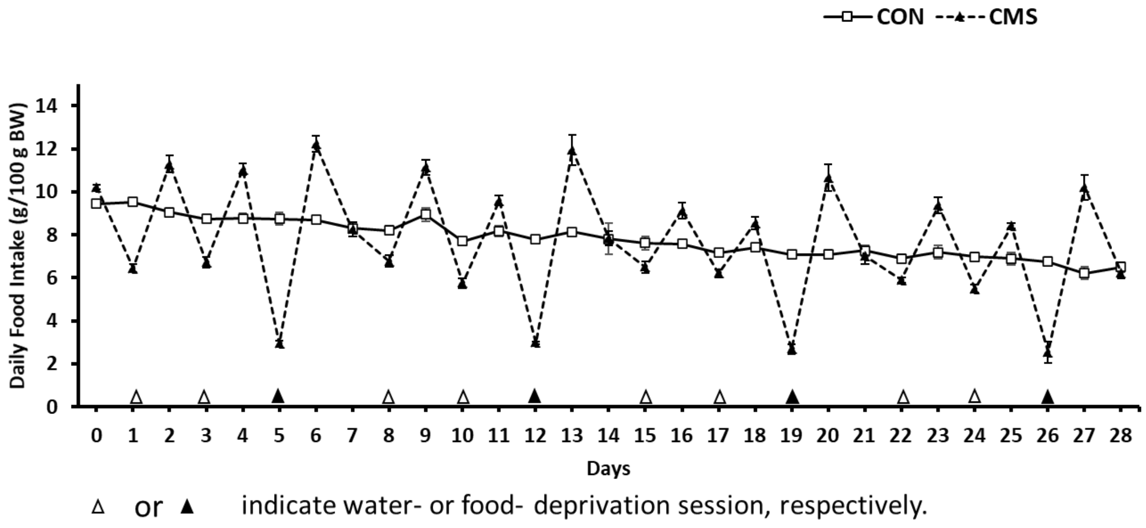

The food intake in the CMS was increased following food or water deprivation session of the previous day suggesting the compensatory increased in food intake. Therefore, the average food intake was not significant different between control and CMS.

Table S2. Sucrose intake of control and CMS rats during CMS period.

Sucrose intake (g/100 g BW)	Week of CMS						p value		
	**-1**	**0**	**1**	**2**	**3**	**4**	**CMS**	**Week of CMS**	**CMS×week**
Control	1.67±0.06	1.65±0.05	1.38±0.12	0.97±0.14$^{\#\#\#}$	0.97±0.09$^{\#\#\#}$	1.14±0.07$^{\#\#}$	0.001	< 0.001	< 0.001
CMS	1.05±0.10***	0.99±0.04***	1.21±0.12	1.01±0.10	0.83±0.10	1.10±0.11			

Data are presented as mean ± S.E.M. *** p < 0.001 vs. control at the same time point, $^{\#\#}$ P < 0.01, $^{\#\#\#}$P < 0.001 vs. baseline (week -1) within group, Two-way repeated measures ANOVA followed by Dunnett's; n=7 for each group.

Table S3. Time and frequency domain parameters of HRV in control and CMS (non-normalized data).

Parameters	Group	Week of CMS					p value		
		0	1	2	3	4	CMS	Week of CMS	CMS×week
**Time Domain**									
RR interval (ms)	Control	124.39±2.62	122.69±2.41	129.47±2.92	132.24±4.07	132.14±5.88	0.452	0.007	0.454
	CMS	118.86±3.23	124.49±4.26	127.64±4.15	131.43±3.27$^{\#}$	123.99±2.00			
HR (bpm)	Control	484.80±9.71	491.06±9.88	467.00±9.99	456.97±13.04	460.47±19.20	0.445	0.004	0.521
	CMS	508.11±13.07	486.30±15.53	473.86±14.96	459.64±11.50$^{\#}$	485.29±7.91			
SDNN (ms)	Control	6.23±0.59	5.23±0.36	7.26±0.59	5.69±0.53	7.09±0.95	0.418	0.610	0.010
	CMS	5.74±0.61	5.86±1.00	5.24±0.44	6.98±0.92	4.61±0.52*			
RMSSD (ms)	Control	3.14±0.32	3.18±0.15	2.98±0.21	3.59±0.34	3.23±0.35	0.477	0.064	0.675
	CMS	2.62±0.20	2.75±0.09	2.81±0.19	3.60±0.35	3.48±0.47			
PNN10 (%)	Control	1.28±0.32	1.76±0.26	0.64±0.18	2.21±0.85	1.57±0.73	0.806	0.074	0.400
	CMS	0.40±0.20	0.47±0.10	0.65±0.28	2.32±0.73	3.03±1.55			
**Frequency Domain**									
VLF (%)	Control	85.18±2.46	81.47±2.06	83.98±2.29	79.25±2.13	83.52±2.13	< 0.001	0.362	< 0.001
	CMS	86.80±2.45	81.78±2.83	85.44±0.96	82.08±1.87	66.50±4.49$^{***,\#\#\#}$			
LF (%)	Control	6.05±0.73	6.01±0.52	6.79±0.54	6.19±1.15	6.06±0.80	0.118	0.070	0.031
	CMS	5.89±0.96	7.55±1.44	6.16±0.58	8.25±0.94	11.55±1.58$^{*\#}$			
HF (%)	Control	6.99±1.45	9.82±1.30	7.97±1.93	10.70±1.47	8.40±1.28	0.777	< 0.001	0.001
	CMS	5.86±1.27	8.47±1.59	6.74±0.78	7.90±0.93	16.99±2.88$^{***,\#\#\#}$			
LF/HF ratio	Control	1.03±0.17	0.67±0.07	0.99±0.21	0.65+0.16	0.78±0.10	0.244	0.125	0.290
	CMS	1.06±0.12	0.96±0.14	1.01±0.16	1.07±0.10	0.76±0.13			

Data are presented as mean ± S.E.M. * $p < 0.05$, *** $p < 0.001$ vs. control at the same time point, $^{\#}$ $p < 0.05$, $^{\#\#\#}$ $p < 0.001$ vs. baseline (week 0) within group, Two-way repeated measures ANOVA followed by Dunnett's; n=7 for each group.
